# A Cross-Day Analysis of EMG Features, Classifiers, and Regressors for Swallowing Events Detection and Fluid Intake Volume Estimation

**DOI:** 10.3390/s23218789

**Published:** 2023-10-28

**Authors:** Iman Ismail, Imran Khan Niazi, Heidi Haavik, Ernest N. Kamavuako

**Affiliations:** 1 Department of Engineering, King’s College London, London WC2R 2LS, UK; iman.a.ismail@kcl.ac.uk; 2 Centre for Chiropractic Research, New Zealand College of Chiropractic, 6 Harisson Road, Mount Wallington, Auckland 1060, New Zealand; imran.niazi@nzchiro.co.nz (I.K.N.); heidi.haavik@nzchiro.co.nz (H.H.); 3 Faculté de Médecine, Université de Kindu, Site de Lwama II, Kindu, Maniema, Congo

**Keywords:** dehydration, electromyography sensors, EMG features, fluid intake, drinking, classification, fluid estimation

## Abstract

Dehydration is a common problem among older adults. It can seriously affect their health and wellbeing and sometimes leads to death, given the diminution of thirst sensation as we age. It is, therefore, essential to keep older adults properly hydrated by monitoring their fluid intake and estimating how much they drink. This paper aims to investigate the effect of surface electromyography (sEMG) features on the detection of drinking events and estimation of the amount of water swallowed per sip. Eleven individuals took part in the study, with data collected over two days. We investigated the best combination of a pool of twenty-six time and frequency domain sEMG features using five classifiers and seven regressors. Results revealed an average F-score over two days of 77.5±1.35% in distinguishing the drinking events from non-drinking events using three global features and 85.5±1.00% using three subject-specific features. The average volume estimation RMSE was 6.83±0.14 mL using one single global feature and 6.34±0.12 mL using a single subject-specific feature. These promising results validate and encourage the potential use of sEMG as an essential factor for monitoring and estimating the amount of fluid intake.

## 1. Introduction

Dehydration affects older adults and can have detrimental effects on their health [1]. Older adults may feel less thirsty, which can make them drink less fluid since their bodies are less effective at maintaining fluid balance [2]. Additionally, they may be more prone to conditions that increase their risk of dehydration, such as kidney disease, diabetes, and certain medications [3]. They face considerable hydration concerns since their bodies contain 10 to 15% less water which may lead to many health problems [4]. A review revealed that most older adults are more susceptible to renal issues and electrolyte abnormalities due to medications that lead to dehydration, making them more susceptible to changes in conditions and illnesses [4].

Fluid charts are one of the crucial clinical tools used in hospitals and care facilities where nurses keep an eye on the consumption of meals and liquids to track patients’ fluid intake and output throughout the day [5]. However, fluid charts have limitations because nurses sometimes fail to note a patient’s intake [6]. According to Asogan (2021), only 25% of the fluid charts at Kettering General Hospital had precise measurements, and only 14% had thorough records of all intakes and outputs [7]. Therefore, there is a need to develop technologies like wearable technologies for accurate fluid intake estimation and monitoring to reduce the risk of dehydration.

There is a growing interest in the potential of wearable technologies to monitor various aspects of health, including fluid intake [8]. Wearable devices may facilitate real-time monitoring and tracking of fluid intake. Many wearable technologies have been used to monitor fluid intake, such as accelerometers, inertial sensors, smartwatches, cellphones, acoustic sensors, and electromyography sensors [9,10,11,12,13,14]. These items are widely available on the commercial market and have helped to identify drinking activities like drinking from different containers (cups, bottles, straws, and glass) and continuous or discrete volumes of fluid [6,15]. However, they cannot reliably estimate the fluid volume, despite research showing that they can detect drinking events using machine learning with an accuracy of >80%. Additionally, many older adults dislike these devices and do not wish to wear them [16]. Another approach to measuring fluid intake is using smart surfaces with embedded sensors [17]. These surfaces are impractical as they require the users to lift the containers used for drinking and place them on the surface every time they drink to determine the drinking actions and record the drink amount [18]. Any additional object placed on the surface or spilling the content in a sink will give inaccurate information, leading to erroneous detections. Furthermore, some vision and environmental approach techniques, like wearable cameras and radar, have focused only on intake detection [8]. However, the detection accuracy depends on the camera resolution and the surrounding environment (lighting, processing power, and data storage), most of which have yet to be operated in real time. Nevertheless, these techniques can recognise drinking events with close to 90% accuracy using deep learning techniques, but determining whether fluid has been consumed and estimating the volume of fluid consumed remains challenging [11,19,20,21].

Some techniques are based on physiological signals to monitor fluid intake, including surface electromyography (sEMG) sensors. The use of physiological sensors to capture swallowing events is based on the fair assumption that fluid consumption can only be confirmed after it has been swallowed. Malvuccio and Kamavuako (2021) applied sEMG recordings of individual and continuous swallows to distinguish between liquid and saliva swallows using fine K-nearest neighbour (KNN) with an accuracy of 86.7±5.52%. Additionally, they achieved an accuracy of 99.0±1.30% in classifying between the noise and swallows using fine Gaussian support vector machine (SVM) [6]. They also investigated the effect of sEMG features on classifying the swallowing events and estimating fluid volume [21]. Ismail and Kamavuako estimated the volume of the fluid intake using sEMG with a root mean square error of 1.37±1.10 mL using random forest (RF) and one feature [22].

Surface EMG and microphones were used to continuously monitor swallowing events by Amft and Tröster to discriminate between solid and liquid meals in a single participant [23]. Nicholls et al. (2022) used EMG to detect eating behaviour and combined it with real-time wristband haptic feedback to facilitate mindful eating. A support vector machine was used for chewing classification, with an F-score of 0.95 and for swallowing classification, with an F-score = 0.87 [24]. Vaiman et al. (2003) used EMG to make a database for the duration and amplitude of muscle activities of 100 children during swallowing and continuous drinking which can be used to detect abnormalities in pediatric patients and provide a basis for comparison of swallowing performance, both within and between patients [25]. Nederkoorn et al. (1999) measured the swallowing activity using (EMG) to assess the amount of saliva secreted using the number of peaks in the EMG activity of the musculus digastricus [26]. Vinyard et al. (2016) used EMG to study the relationships between food textures and oral processing. In this study, food scientists used EMG from the feeding muscles as (1) a general measure of food texture, (2) a measure of oral physiology, (3) an estimate of absolute force, and (4) a measure of muscle work [27]. Another study integrated the EMG sensor into wearable glasses to measure temporalis muscle activity to detect intake-related events. It achieved 96% accuracy for counting the number of chewing cycles and up to 90.8% accuracy for classifying five types of food [28]. sEMG has been used in some studies to monitor food and fluid. However, its sole use to detect fluid-swallowing events from a mixed pool with solid and saliva swallows and cough has not been attempted before and the number of studies that estimate the volume remains limited.

The challenge is not only to classify the liquids but also to estimate the volume of the fluid intake. Therefore, this study aims to compare the effect of sEMG features on the classification and estimation of the volume of fluid intake. Novel contributions of this paper include (1) investigating the optimal subset of EMG features in classifying the drinking events (from a mixed pool with solid, saliva, and cough) and in estimating the fluid intake volume; (2) unravelling the dependency between the choice of classifier/regressor and features; and (3) exploring cross-day data for classification and regression for fluid intake quantification. To the best of our knowledge, this is the first study investigating the combination of optimum feature classifiers across days for swallowing detection and fluid volume estimation from sEMG.

This paper is organised so the Section 2 provides the experimental approach and data analysis. Section 3 separates classification results from estimation results. Each subsection breaks the results into single days and cross-days for clarity in both cases. Section 4 discusses the results and provides the conclusion.

## 2. Methodology

### 2.1. Subjects

This study was conducted in accordance with the declaration of Helsinki and was approved by The Research Ethics Panel of King’s College London (LRS-18/19-10877). Eleven individuals (nine females and two males), aged 20 to 59 years (median age 25 y), participated in the study with no known pre-existing medical conditions and with normal skin turgor. Each participant provided written consent after receiving comprehensive information about the study.

### 2.2. Experimental Procedure

**Figure 1:**
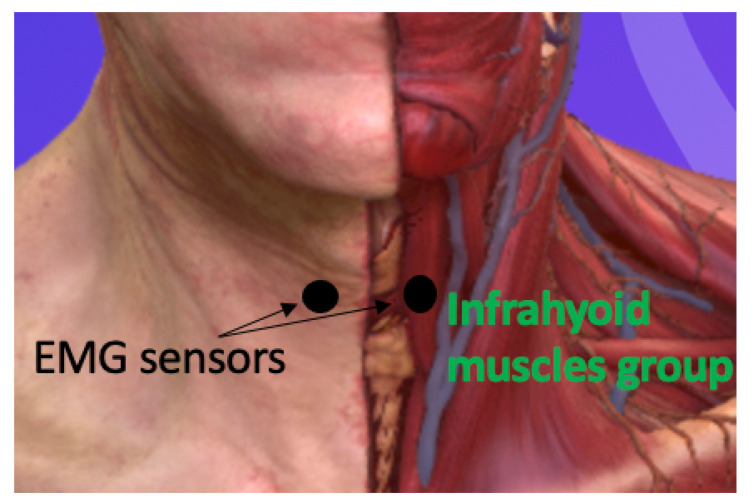
Representation of the anatomical position of the infrahyoid muscles where the Delsys sensors were positioned on the neck, modified from an anatomical software [29].

The experiment involved two sessions, each lasting 90 min, scheduled on two consecutive days. Two Delsys Trigno sEMG sensors (Natick, MA, USA) were used to capture sEMG. sEMG was analogue filtered between 10 and 850 Hz and sampled at 2.2 kHz. The sensors were positioned on both sides (left and right) of the sternohyoid muscles’ belly, which is part of the infrahyoid group. The choice of the sternohyoid muscles was based on their superficial location, and the choice of two sensors was motivated by our previous study [22], where four sensors did not improve performance. Participants were comfortably seated, and the skin in the neck area was cleaned using alcohol wipes. The placement of the sensors was determined by palpating the relevant swallowing muscles, as shown in Figure 1. Once the sensors were correctly positioned anatomically, participants were given verbal cues to perform nine different tasks. The order of the tasks was randomised for each session. The first task involved participants pronouncing ten words while being recorded. Participants were asked to cough for the second task, while the third and fourth tasks involved swallowing saliva and solid food. Solid food provided to participants was chocolate chip cookies, and they were instructed to take one bite at a time. Tasks five through nine focused on participants swallowing water from a cup in a single sip, with the volume of water gradually increasing by 5 mL for each task. The starting volume for the fifth task was 5 mL, and it increased incrementally until reaching 25 mL in the ninth task. A needleless syringe with markings was used for accurate measurement. Overall, participants performed these tasks following verbal instructions, and their actions were recorded for analysis and further evaluation.

### 2.3. Data Analysis

Data analysis was carried out on Google Collab using Python 3.8 and preprocessed using bandpass filtering between 10–400 Hz. Data were analysed for each individual subject (intrasubject analysis). EMG signals were rectified, and the signal envelope was computed to detect the highest peak where the swallowing event occurred. The EMG burst was then extracted using the peak position. The region of the burst was located by identifying the highest value of the peak involving 1 s before and after the highest peak, resulting in a total duration of 2 s. Twenty-six time and frequency domain features (Table 1) were calculated from the raw data of that burst window, representing a single sip. A binary classification for drinking events (swallowing fluid) vs. other events (swallowing solid or saliva, coughing, and talking) was performed using single features and the combination of two to four features to determine the subject-specific best features and global features (across subjects). Carlotta and Kamavuako [21] used stepwise forward selection over 46 features to detect swallowing events and demonstrated that performance reached a plateau around four features. This motivated the choice of four features in this study. Subject-specific means the set of features that maximise performance for a specific subject, while global features means the set that maximises the average performance across subjects.

Five classifiers were used in this study: support vector classifier (SVC), random forest (RF), K-nearest neighbour (KNN) with k = 1, linear discriminant analysis (LDA), and quadratic discriminant analysis (QDA). F-score was used as a performance metric due to the imbalanced sample sizes between the classes. Each subject’s data had 60 drinking samples and 40 nondrinking samples. The k-fold-cross-validation method was used in the classification models with five folds. Thus, all the samples were randomly split into five folds. For each iteration, one subset is designated as the test/validation set, while the remaining four subsets are combined to create the training set. The result is then calculated for each trial and averaged across all five trials to determine the overall effectiveness of our model.

We tested eight regressors: support vector regressor (SVR), random forest (RF), K-nearest neighbour (KNN) with k = 1, linear regressor (LR), decision tree (DT), lasso, ridge, and artificial neural network (ANN) with two hidden layers with thirty-six neurons and sixteen neurons, respectively. The root mean square error (RMSE) was used as the performance metric. The data for each day and each feature type (global features vs. subject-specific features) were analysed separately. The combination of the best features from both sides was also tested. In cross-days analysis, a twofold validation procedure was used with data from days 1 and 2 with the optimum subset of features, best classifiers, and regressors.

For each day and feature type (subject-specific vs. global), a three-way analysis of variance (ANOVA) test was used to test if there were significant differences between the number of features, between the classifiers, and between the right and left side sEMG electrodes. A two-way ANOVA test was used to test if there were significant differences between the regressors and the right and left sides of the sEMG electrodes.

## 3. Results

**Figure 2:**
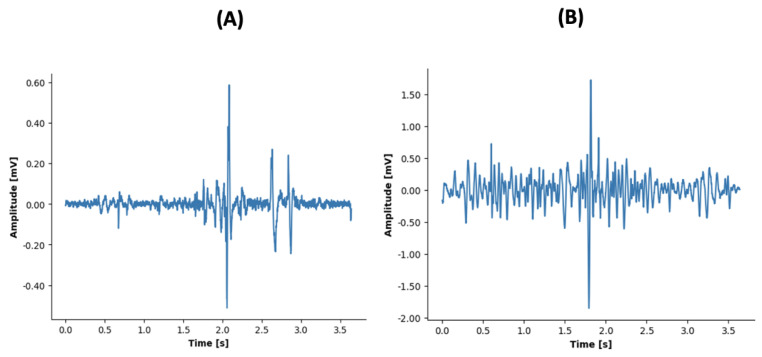

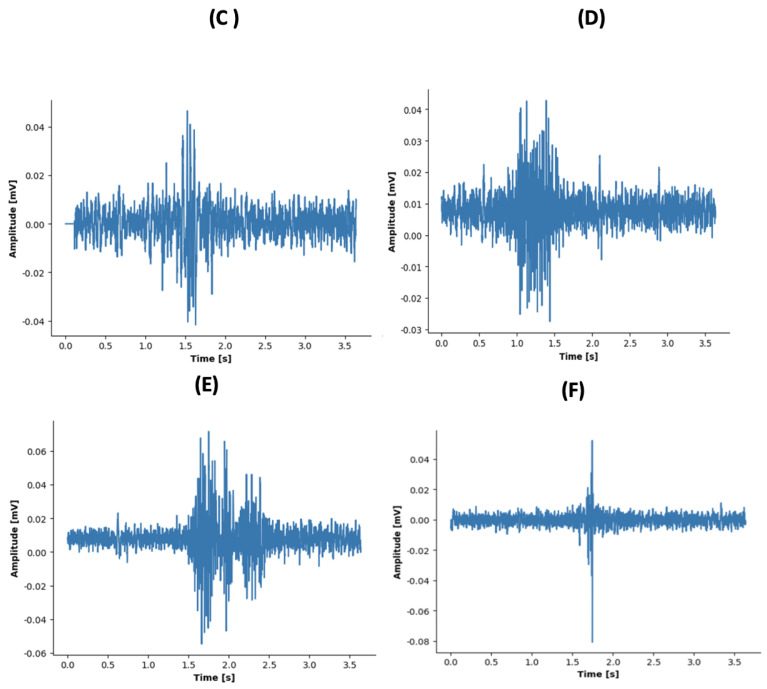
Representation of raw sEMG data of swallowing (**A**) 10 mL of water, (**B**) 25 mL of water, (**C**) saliva, (**D**) solid, (**E**) cough, and (**F**) talk.

Swallowing events were successfully recorded using the EMG sensors for all subjects. Figure 2 depicts raw EMG data recorded for one subject for two different volumes of water, 10 mL and 25 mL, and other nondrinking activities like saliva, solid, cough, and talk. The responses to swallowing the two volumes exhibit different characteristics.

### 3.1. Classification

**Figure 3:**
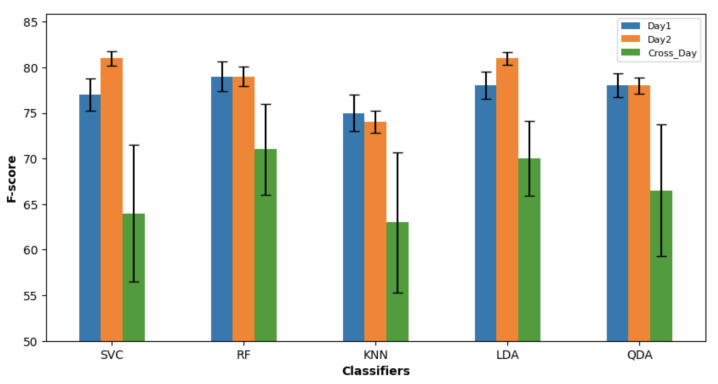
Classifiers performance with error bars depicting for the F-score and standard error over day 1, day 2, and cross-day.

Overall, LDA and RF showed good performance for each day and cross-days, as shown in Figure 3. There was no difference between the right and left EMG channels. Three features showed overall good performance.

#### 3.1.1. Day 1

**Figure 4:**
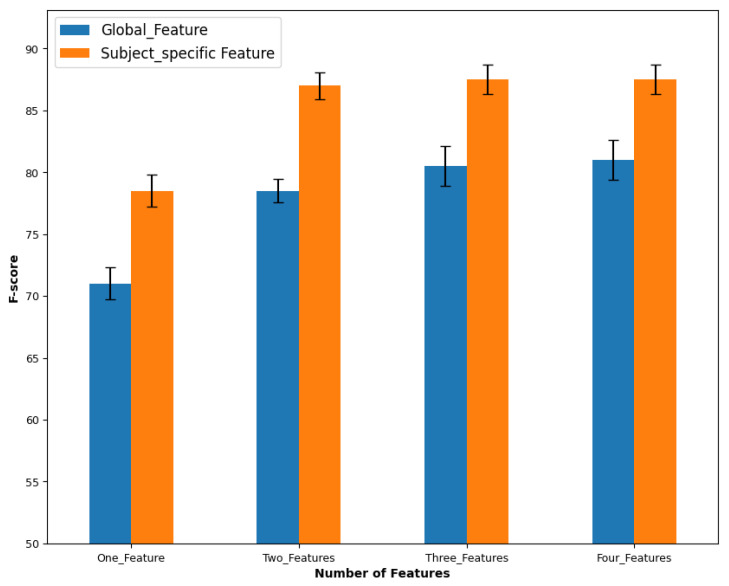
The average F-score of the performance of each number of features of day 1 and day 2.

**Figure 5:**
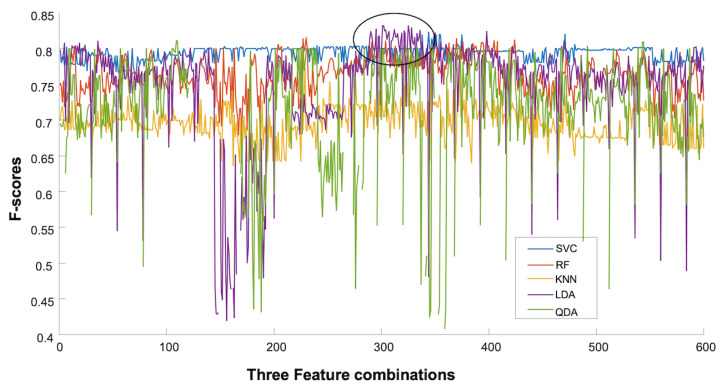
The performance of the three features’ combination with all the classifiers of the right side of day 1. The rounded area in black indicates that only a limited number of sets provide good performance for the LDA.

Using global features, the ensemble mean F-score for day 1 was 77%±2, with the right side (78%±1) performing similarly (*p* = 0.39) to the left side (76%±2). A significant difference was found between classifiers (*p* = 0.02) and the number of features (*p* = 0.001). The best three classifiers were LDA (79%±2), QDA (79%±2), and RF (78%±1). Three features (80%±1.8) performed better than one and two, as shown in Figure 4. There was an interaction between sides and classifiers (*p* < 0.001), between sides and number of features (*p* = 0.004), and between sides, classifiers, and number of features (*p* = 0.04). Figure 5 depicts the variation in performance of the combination of three features across different classifiers.

For subject-specific features, the global mean F-score for day 1 was 84%±1, with the right side (84%±2) performing the same (*p* = 0.64) as the left side (84%±1). A significant difference was found between classifiers (*p*≤ 0.001) and between the number of features (*p* < 0.001). The best three classifiers were RF (86%±1), LDA (85%±1), and QDA (85%±1). Three features (86%±2) performed significantly (*p* < 0.001) better than the two features (85%±1) and the one feature (78%±2). There was an interaction between sides and the number of features (*p* < 0.001), and between sides, classifiers, and the number of features (*p* = 0.02).

However, it can be seen from Figure 5 that their performance is feature-set-dependent, especially the LDA. On the other hand, the SVM showed robustness over changes in the feature set and could be explored more in future studies, allowing for the selection of features with low computation costs.

#### 3.1.2. Day 2

Global features produced an ensemble global mean F-score for day 2 of about 78%±0.7. Four features (83%±1) and the three features (82%±1) performed significantly (*p* < 0.001) better than the single and the two features. The best three classifiers were LDA (81±0.7%), SVC (81%±0.8), and RF (79%±1). In contrast to day 1, there was no interaction between factors.

On the other hand, subject-specific features resulted in a global mean F-score for day 2 of 87%±1. The best three classifiers were LDA (88%±1), QDA (88%±1), and RF (88%±0.9). In contrast to day 1, there was no difference between the number of features (*p* = 0.51). For example, four features gave 89%±1, and three features gave 89%±2. There was no interaction between factors. Table 2 depicts the best single-specific features with the best classifiers of day 1 (right side) which shows that the best feature combination is data-dependent and subject-dependent.

In the cross-day section, training was implemented using the best three global features with the best three classifiers of day 1, and then testing was carried out using the data of day 2. Similarly, training was implemented using the best three global features with the best three classifiers of day 2, and then testing was carried out using the data of day 1. The global mean F-score for the two days was (69%±3.95), with no significant difference between the right and the left sides (*p* = 0.87). There was no significant difference (*p* = 0.51) between the best three classifiers, LDA (70%±4.15), RF (71%±4.95), and QDA (66.5%±7.2). Table 3 presents the F-score of the best global features with the best classifiers on the right side of day 1 and day 2.

### 3.2. Estimation

**Figure 6:**
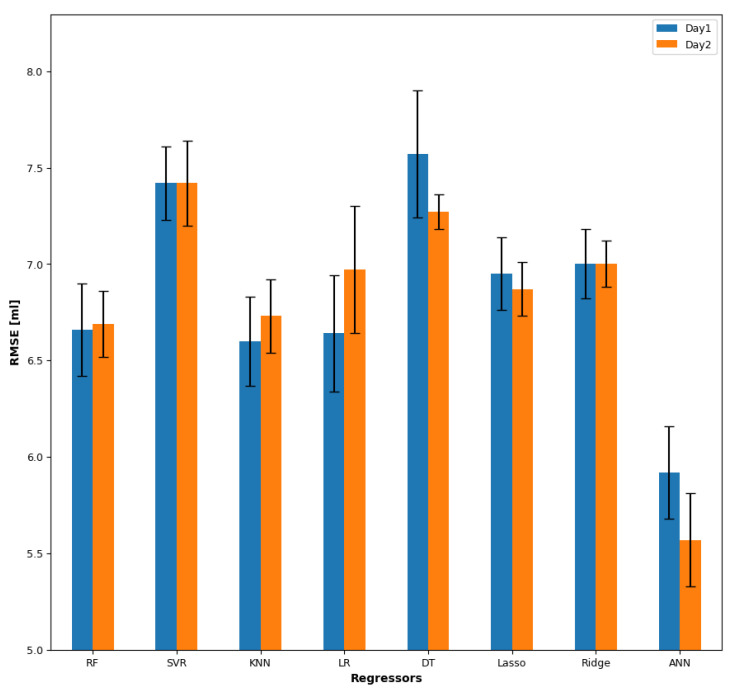
The regressors’ performances with error bars depicting the RMSE and standard error over day 1 and day 2.

Overall, ANN had the lowest absolute RMSE, as shown in Figure 6, and the right side channel performed better than the left side. Using more than one feature did not improve the performance of the regressors.

#### 3.2.1. Day 1

The global mean RMSE of day 1 using one global feature was 6.85 ± 0.18 mL, with the right side (6.33±0.27 mL) performing significantly (*p* = 0.008) better than the left side (7.36±0.2 mL). A significant difference (*p* < 0.001) was found between the regressors ANN (5.92±0.24 mL), KNN (6.6±0.23 mL), and LR (6.64±0.3 mL). The best global features used with the ANN were skew for the right side and LPCC for the left side, respectively. With subject-specific feature, the global mean RMSE of day 1 was 6.31±0.15 mL, with the right side (5.89±0.25 mL) performing significantly (*p* = 0.01) better than the left side (6.7±0.15 mL). A significant difference (*p* < 0.001) was found between the regressors ANN (4.88±0.2 mL), LR (6.09±0.21 mL), and SVR (6.14±0.17 mL). Table 4 illustrates the optimal subject-specific feature paired with the best regressor of the right side for day 1.

#### 3.2.2. Day 2

The global mean RMSE of day 2 using global features was 6.81 ± 0.1 mL, with the right side (6.38±0.15 mL) performing significantly (*p* = 0.007) better than the left side (7.25±0.18 mL). Similar to day 1, there was a significant difference (*p* < 0.001) between the regressors ANN (5.57±0.24 mL), SVR (6.69±0.17 mL), and KNN (6.73±0.19 mL). The best global features used with the ANN were FR for the right side and MDF for the left side.

With subject-specific feature, the global mean RMSE of day 2 was 6.36±0.08 mL, with the right side (5.97±0.14 mL) performing significantly (*p* = 0.004) better than the left side (6.75±0.13 mL). Similar to day 1, there was a significant difference (*p* < 0.001) between regressors ANN (4.62±0.24 mL), SVR (6.26±0.13 mL), and KNN (6.26±0.13 mL).

#### 3.2.3. Cross-Day

In the cross-day analysis, training was implemented using the data and the best global features with the best regressor (ANN) of day 1, and then testing was carried out using the data of day 2. Similarly, training was implemented using the data and the best global features with the best regressor (ANN) of day 2, and then testing was carried out using the data of day 1. The global mean RMSE for the two days was (8.69±1.1 mL), with no significant difference between the right and the left sides (*p* = 0.51).

## 4. Discussion

The results obtained in this study suggest that sEMG signals can be used effectively to distinguish between drinking events and nondrinking events (solid food, saliva, cough, and talk). The performance is acceptable as the nondrinking events also contain swallowing of solid food and saliva, demonstrating the power of indirect classification between drinking and eating and volume estimation with single-channel sEMG across two days. Identifying the optimal global set of features enhances the classification model’s performance instead of using only single features.

The performance of the right side of the infrahyoid muscles was higher in absolute value than the left side, but there were no significant differences between the two sides. Three and four features showed overall higher performance than one and the two features. There was no significant difference between three features and four features for day 1 and day 2; therefore, choosing three features for our implementation will be a good option for computational efficiency and cost-effectiveness, particularly in continuous online tasks. This study revealed that optimum features are subject-dependent and classifier-dependent. Subject-specific features performed better than general features, but their generalisation power needs to be quantified in a real-life environment. The F-score metric was used to evaluate the classification performance due to the imbalance of the sample number. Furthermore, there are other papers that focus on classifying and estimating fluid intake volume using sEMG. Malvuccio et al. (2022) classified the liquid swallows from nonliquid swallows using five features and two sEMG channels. However, our study used only three features and only a single sEMG channel. Malvuccio and Kamavuako detected noise from swallows and classified saliva from other liquid swallows [21]. This study classified liquid classes; however, our study detected liquid swallows from nonliquid swallows.

For the regression, the study suggests that estimating fluid volume intake is feasible using sEMG. This study showed how regression performance differed depending on the regressor and feature. ANN produced the best performance for the estimation with only a single feature. Increasing the number of features did not improve the results compared to the classification. The right side channel performed better than the left side. Estimation results were poorer than previous studies [6,22], potentially because the recorded amounts of drinking were discreet: 5 mL, 10 mL, 15 mL, 20 mL, and 25 mL. Previous studies used self-controlled volumes dictated by the swallowing capacity of the subjects [6,22]. This study revealed that no single regressor achieves optimal performance across all features, suggesting that the choice of regressor depends on the specific feature being considered. Consequently, utilising a single feature can offer advantages in terms of reducing computational expenses and saving time, particularly in the context of online tasks. Nevertheless, we aim to explore the use of neural networks in future studies focusing on architecture and hyperparameter optimisation.

Moreover, the number of studies focusing on fluid volume estimation from sEMG is very limited. Kobayashi et al. used a throat microphone to measure the amount of liquids consumed, with an RMSE value of 3.33 mL [21]. Malvuccio also estimated the amount of fluid consumed using sEMG recordings of both individual and continuous swallows, with RMSE of 2.80±1.22 mL [21], using recordings of single swallows with RMSE value (2.01±1.39 mL), and recordings of continuous swallows with RMSE of (25.82±26.39 mL) [6]. Ismail and Kamavuako estimated the fluid intake volume, using sEMG with an RMSE of 1.37±1.1 mL, using only a single feature [22]. In the previous study [22], utilising the mean absolute value (MAV) as the best average feature for fluid volume estimation and employing an ANN resulted in a poorer performance than previous studies. Our conclusion is that the best approach for volume estimation is to let the user drink to the best of their ability, and this will be the approach we take in all future studies. Imposing the volume (5 mL to 25 mL) forces the user to drink unnaturally, which might affect muscle activation. In this study, different window sizes (from 0.1 s to 2 s) were tested to see the effect of changing the window size on our results of classification and estimation. The results indicated that there is no need for larger windows, and the window size can be reduced to 0.5 s or 1 s without degradation in performance.

### Limitations of the Study

Despite achieving good and comparable performance, there is room for improvement in reducing errors and increasing the system’s performance. It would be beneficial to make further improvements in further research, such as larger sample sizes and deep neural networks, including real-time detection and estimation. Additionally, we expected to observe symmetry in the classification and regression results of the right side and the left side; however, the results revealed that the performance of the right side is higher than the left side, which may be due to the difference in electrode placement. The left electrodes may not have been positioned properly on the swallowing muscles, and may have slid over the skin. Electrode placement should be considered carefully in the future, by possibly using lighter electrodes such as Delsys Trigno minisensors. Furthermore, participants in this study are relatively young compared with potential users of such a system. Although we provided proof of concepts in normal skin conditions, future studies should consider including older adults, where skin turgor decreases due to ageing. Skin turgor should be considered with older adults as the interface between electrode–skin–muscle might change, affecting the quality of the EMG.

## 5. Conclusions

The findings of this study indicate that sEMGs (surface electromyograms) can differentiate between fluid and nonfluid swallowing events, as well as provide some degree of fluid intake volume estimation by utilising an optimal feature set. The LDA (linear discriminant analysis) demonstrated strong performance in detection using three features, while the ANN (artificial neural network) excelled in volume estimation, particularly when utilising the right sEMG channel. These results are a step forward in developing a noninvasive device for effectively monitoring fluid intake, thereby enhancing the health and care system.

## Figures and Tables

**Table 1:** This table presents the twenty-six features included in this study, maintaining the same order in which they were computed.

Feature Full Name	Abbreviation	References
Autoregressive Coefficients	AC	[30,31]
Avergare Amplitude Change	AAC	[32,33]
Calc Centroid	CC	[34]
Different Absolute Standard Deviation Value	DASDV	[32,33]
Entropy	MYop	[35]
Empirical Cumulative Distribution	ECDF	[34]
Frequency Ratio	FR	[33]
Linear Prediction Cepstral Coefficients	LPCC	[34]
Log Detector	LOG	[30,36]
Kurtosis	Kurt	[37,38]
Mean Power	MNP	[33,39]
Mean Frequency	MNF	[39,40]
Median Frequency	MDF	[33,39]
Mean Absolute Value	MAV	[41,42]
Myopulse Percentage Rate	MYOP	[33]
Mel Frequency Cepstral Coefficients	MFCC	[43]
Peak Frequency	PKF	[33]
Power Spectrum Density Bandwidth	PW	[36]
Skewness	Skew	[38,42]
Spectral Centroid	SC	[34]
Spectral Entropy	SE	[34]
Spectrogram Frequency	SF	[34]
Variance	VAR	[30,31]
Wavelength	WL	[30,41]
Willison Amplitude Change	WAMP	[30,31]
Zero Crossing Rate	ZC	[30,41]

**Table 2:** The F-score of the best subject-specific feature with the best classifiers of day 1 (right side).

Subjects	1F	2F	3F	4F
S1	80% (LogD) SVC, QDA	85% (Kurtosis, PW) RF	84% (LogD, WL, FR) (MAV, LogD, PW) RF, QDA	86% (PW, CC, Kurt, AAC) RF
S2	80% (MNF) (MDF) (AC) LDA	99% (MNP, MNF) (FR, MNP) RF, SVC, KNN, LDA	99% (Fr, MP, MF) (MP, MF, MDF) (Fr, MP, MF) (FR, MNP, SF) RF, SVC, KNN, LDA, QDA	99% (FR, MNP, MNF, MDF) (MNP, MNF, MDF, PF) (FR, MNP, MNF, MDF) RF, SVC, KNN, LDA
S3	77% (MAV) (LogD) (MAV) (WL) (AAC) SVC, QDA, LDA	82% (SC, Skew) (AC, DASD) RF, LDA	91% (AC, Entropy, SC) LDA	83% (MDF, PF, AC, MAV) LDA
S4	89% ZC LDA, QDA	93% (MNP, ZC) QDA	96% (MNP, MNF, ZC) QDA	96% (AAC, DASDV, ZC, MNP) QDA
S5	81% SC, CC LDA	83% (MNF, PW) (MFCC, MNP) RF, QDA	96% (MNP, MNF, ZC) LDA	84% (Entropy, ECDF, SC, LogD) LDA
S6	78% SF, SVC	87% (myop, LPCC) LDA	89% (MDF, PF, myop) SVC	86% (FR, MNP, MNF, ZC) QDA
S7	88% Kurt SVC	91% (AC, SC) LDA	91% (AC, myop, ECDF) LDA	94% (PW, CC, Kurtosis, AAC) RF
S8	88% AC QDA	89% (myop, Kurt) SVC	94% (kur, skew, WL) RF	94% (LogD, WL, AAC, PW) RF
S9	77% myop SVC	83% (MNP, WL) QDA	79% (MNP, MDF, PW) RF	79% (MNP, MNF, MDF, MFCC) RF (DASDV, ZC, WAMP, PW) KNN
S10	73% WAMP RF, LogD, LDA	80% (SF, MDF) RF	83% (MF, MDF, PW) LDA	80% (WAMP, myop, VAR, Kurt) LDA (WAMP, myop, VAR, PW) QDA (DASDV, ZC, WAMP, PW) KNN
S11	88% AC QDA	83% (MNP, myop) LDA	83% (myop, VAR, MNP) LDA	92% (PF, AC, myop, MDF) LDA

**Table 3:** A summary of the F-score of the best global features with the best classifiers of the right side of day 1 and day 2.

–	Number of Features	Best Global Features	F-Score	Best Calssifiers
Day 1	1F	AAC	74%±2	RF
	2F	LPCC, MAV	83%±1.9	LDA
	3F	LPCC, MAV, LogD	83%±1.9	LDA
	4F	LPCC, MAV, LogD, WL	83%±2.1	LDA
Day 2	1F	FR	69%±1.3	SVC
	2F	LogD, WAMP	82%±1.7	SVC
	3F	LPCC, MAV, AAC	84%±1.7	LDA
	4F	SC, SE, SF, ECDF	84%±1.8	SVC

**Table 4:** The RMSE of the best subject-specific feature with the best regressor of day 1 (right side).

Subjects	RMSE	1F
S1	4.67	FR
S2	3.07	MFCC
S3	5.04	Kurt
S4	5	MFCC
S5	4.58	Kurt
S6	2.88	Skew
S7	3.87	FR
S8	5.07	MDF
S9	5.59	ZC
S10	6.05	Kurt
S11	4.94	SF

## Data Availability

Raw data are available for sharing if requested.
